# Appendiceal GIST: report of an exceptional case and review of the literature

**DOI:** 10.11604/pamj.2013.15.85.2430

**Published:** 2013-07-03

**Authors:** Mahdi Bouassida, Mohamed Fadhel Chtourou, Emna Chalbi, Fathi Chebbi, Lamine Hamzaoui, Selim Sassi, Lamia Charfi, Mohamed Mongi Mighri, Hassen Touinsi, Adok Sassi

**Affiliations:** 1 Department of surgery, Mohamed Tahar Maamouri Hospital, Nabeul, Tunisia; 2 Department of cytology, Mohamed Tahar Maamouri Hospital, Nabeul, Tunisia; 3 Department of gastroenterology, Mohamed Tahar Maamouri Hospital, Nabeul, Tunisia

**Keywords:** Appendiceal GIST, peritonitis, surgery, CD34

## Abstract

Gastro-intestinal stromal tumors (GISTs) of the appendix are a rare entity. To date, only eight cases has been described in the literature, most of which have been of the benign type. We report a new case of an appendiceal GIST in a 75-year-old man. The tumor was discovered when the patient presented with acute appendiceacal peritonitis. Preoperative diagnosis of appendiceal GIST was rarely done as tumors were usually associated with appendicitis-like symptoms.

## Introduction

Gastrointestinal stromal tumors (GIST) are the most common primary mesenchymal neoplasms of the gastrointestinal (GI) tract. GISTs occur most commonly in the stomach (60%) and the small bowel (30%). Appendiceal GISTs are extremely rare making up approximately 0.1% of all cases, with eight cases reported in the literature thus far, seven of these cases were benign. Only in one reported case, the malignant nature of the lesion was confirmed.

## Patient and observation

A 75-year-old man was brought to the emergency department with a 48-hour history of abdominal pain, emesis, and low fever. He reported that the pain was progressive in nature. The patient′s medical history was significant for hypertension. On physical exam, he was febrile (38.5°C), with normal vital signs. Physical examination demonstrated diffuse lower abdominal tenderness with peritoneal signs. Laboratory tests showed leukocytosis (WBC: 16.000/ mm^3^) and elevation of the C-reactive protein (CRP: 22 mg/dl). There were no other abnormal values on peripheral blood analysis or serum biochemical analysis. Sonography found a noncompressible distended appendix in the right lower quadrant and free fluid in the hole abdominal cavity.

**Figure 1:**
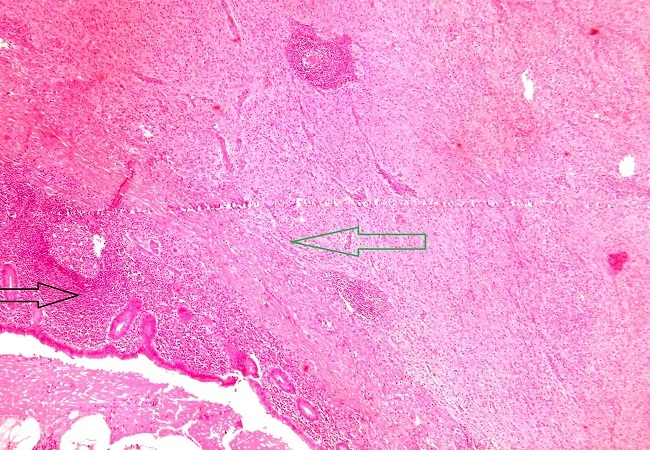
Histopathologic findings: transition area between appendiceal cells (black arrow) and tumoral cells (green arrow) (X200)

**Figure 2:**
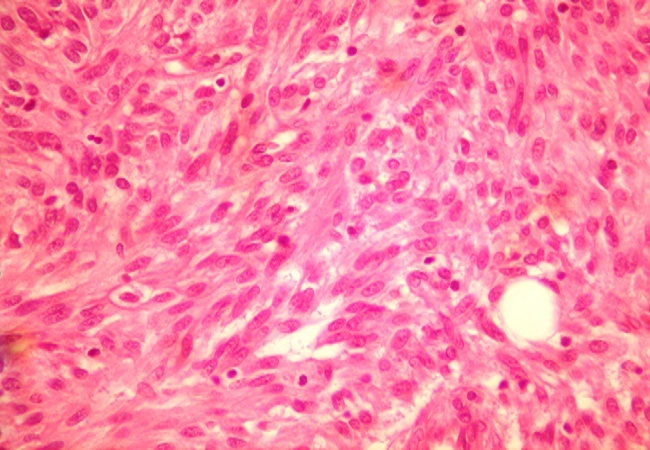
Histopathologic findings: monotonous spindle morphology with irregular fascicles and variably sclerosed stroma (X400)

**Figure 3:**
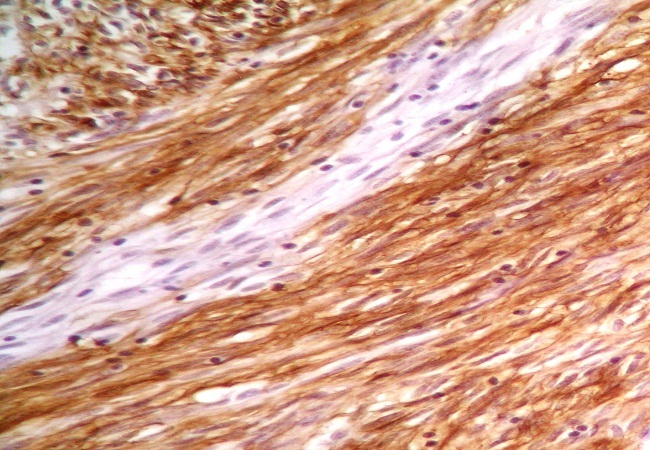
Immunohistochemical findings. Positive staining with CD34

After a pre-operative reanimation, a median laparotomy was performed. The exploration showed an acute generalized peritonitis with 300 mm^3^ of pus, false membranes and a phlegmonous pelvic appendix perforated in its apex. An appendectomy and peritoneal toilet were subsequently performed. A 2 cm nodular protrusion in the mid portion of the appendix was observed, and the specimen was submitted to frozen section. The cut section of the tumor was tan-yellow with vague whorls. Histology showed a monotonous spindle morphology characteristic of GISTs, with irregular fascicles and variably sclerosed stroma (Figure 1, Figure 2). The tumor showed strong expression of CD34 (Figure 3) and CD117 but was negative for desmin, h-caldesmon, SMA, and S-100. The tumor was diagnosed as low-risk GISTs according to the National Institutes of Health consensus criteria for diagnosis and risk assessment of GISTs, corresponding to prognostic groups 2 in the classification by Miettinen and Lasota. Adjuvant therapy was not administrated to the patient and no evidence of recurrence or metastasis was observed at the 48-month follow-up.

## Discussion

Gastrointestinal stromal sarcomas (GISTs) are the most common mesenchymal tumours originating in the digestive tract. They have a characteristic morphology, are generally positive for CD117 (c-kit) and are primarily caused by activating mutations in the KIT or PDGFRA [1, 2] They are located typically in the submucosa of the stomach and the small and large intestines, although cases arising in the esophagus, greater omentum, and mesenterial adipose tissue have been described [3].

Appendiceal GISTs are extremely rare making up approximately 0.1% of all cases [4], with eight cases reported in the literature thus far; seven of these cases were benign and measuring less that 3 cm in diameter [5]. Only in one reported case, the malignant nature of the lesion was confirmed [6].

Based on the previously reported 8 cases and our case, patients with appendiceal GISTs have a mean age of 67 years (range, 56-78 years) with a remarkable predilection for men (3.5:1).

Three tumors were associated with appendicitis-like symptoms in the absence of histologic evidence of acute appendicitis, suggesting that the symptoms were caused by the tumor [5, 7, 8]. Three other tumors were found incidentally during surgery for other diseases or at autopsy [5, 7]. One other tumor was associated with acute appendicitis [7], and with a peri-appendiceal abscess for the last case [6]. For our case, the tumor was discovered when the patient presented with an acute peritonitis.

Consistent with the high incidence of associated other malignancies in GIST patients in general [4], 4 of 9 patients with appendiceal GISTs were affected by other cancers: 3 had carcinomas[5, 7], and 1 had a synchronous malignant gastric GIST [7]. One patient had neurofibromatosis type 2 [8]. The locations were as follows: mid portion (n = 5), tip (n = 3), and proximal part (n = 1). Three tumors were extramural, and 1 was pedunculated. Their mean size was 33.5 mm (range, 2.5- 200 mm). Seven of the eight previously reported appendiceal GISTs were spindled, mitotically inactive lesions corresponding to prognostic groups 1 (n = 6) and 2 (n = 1) according to Miettinen and Lasota [4] and were at very low risk (n = 6) and low risk (n = 1) according to the National Institutes of Health consensus criteria [9]. For one case, the size of the tumor, number of mitosis and locally invasive surgical findings clearly confirm the malignant nature of the lesion [6].

In our case, six previous cases examined by immunohistochemistry showed a uniform coexpression of CD117 and CD34. None was positive for desmin, smooth muscle actin, or S-100. None of 3 patients with follow-up (mean, 49 months)had evidence of progressive disease.

The main problem with our patient was whether to consider the tumor as ruptured, as the patient was in general peritonitis, or not. In patients with tumoral rupture, it would be acceptable to administer imatinib for more than 3 years. On the other hand, our patient had general peritonitis due to a perforation of the apex of the appendix and the tumor was located in the mid portion, so that, adjuvant therapy was not administrated.

## Conclusion

Gastro-intestinal stromal tumors (GISTs) of the appendix are a rare entity. To date, only eight previous cases have been described in the literature, seven of which have been of the benign type. Preoperative diagnosis was rarely done as tumors were usually associated with appendicitis-like symptoms.
